# Combined pituitary hormone deficiency caused by *PROP1* mutations: update 20 years post-discovery

**DOI:** 10.20945/2359-3997000000139

**Published:** 2019-05-13

**Authors:** Fernanda A. Correa, Marilena Nakaguma, João L. O. Madeira, Mirian Y. Nishi, Milena G. Abrão, Alexander A. L. Jorge, Luciani R. Carvalho, Ivo J. P. Arnhold, Berenice B. Mendonça

**Affiliations:** 1 Universidade de São Paulo Hospital das Clínica Faculdade de Medicina Universidade de São Paulo São Paulo SP Brasil Unidade de Endocrinologia do Desenvolvimento, Laboratório de Hormônios e Genética Molecular LIM/42, Disciplina de Endocrinologia, Hospital das Clínicas da Faculdade de Medicina da Universidade de São Paulo, São Paulo, SP, Brasil; 2 Universidade de São Paulo Hospital das Clínicas Faculdade de Medicina Universidade de São Paulo São Paulo SP Brasil Unidade de Endocrinologia Genética, Laboratório de Endocrinologia Celular e Molecular LIM/25, Disciplina de Endocrinologia, Hospital das Clínicas da Faculdade de Medicina da Universidade de São Paulo, São Paulo, SP, Brasil

**Keywords:** PROP1, combined pituitary hormone deficiency, growth hormone deficiency, short stature

## Abstract

The first description of patients with combined pituitary hormone deficiencies (CPHD) caused by *PROP1* mutations was made 20 years ago. Here we updated the clinical and genetic characteristics of patients with *PROP1* mutations and summarized the phenotypes of 14 patients with 7 different pathogenic *PROP1* mutations followed at the *Hospital das Clínicas* of the University of Sao Paulo. In addition to deficiencies in GH, TSH, PRL and gonadotropins some patients develop late ACTH deficiency. Therefore, patients with *PROP1* mutations require permanent surveillance. On magnetic resonance imaging, the pituitary stalk is normal, and the posterior lobe is in the normal position. The anterior lobe in patients with PROP1 mutations is usually hypoplastic but may be normal or even enlarged. Bi-allelic *PROP1* mutations are currently the most frequently recognized genetic cause of CPHD worldwide. *PROP1* defects occur more frequently among offspring of consanguineous parents and familial cases, but they also occur in sporadic cases, especially in countries in which the prevalence of *PROP1* mutations is relatively high. We classified all reported *PROP1* variants described to date according to the American College of Medical Genetics and Genomics and the Association for Molecular Pathology (ACMG-AMP) guidelines: 29 were pathogenic, 2 were likely pathogenic, and 2 were of unknown significance. An expansion of the phenotype of patients with *PROP1* mutations was observed since the first description 20 years ago: variable anterior pituitary size, different pathogenic mutations, and late development of ACTH deficiency. *PROP1* mutations are the most common cause of autosomal recessive CPHD with a topic posterior pituitary lobe. Arch Endocrinol Metab. 2019;63(2):167-74

## INTRODUCTION

It has been 20 years since Wei Wu in M.G. Rosenfeld’s Research Unit first identified inactivating mutations of the *PROP1* gene in humans with combined pituitary hormone deficiency (CPHD) (1). Until now this has been the most common genetic cause of CPHD (2). Over the past 20 years, our Unit at *the Hospital das Clínicas* has contributed significantly to the knowledge of *PROP1* in CPHD, describing for the first time spontaneous involution of the anterior pituitary and the development of cortisol deficiency (3). We also expanded the spectrum of *PROP1* mutations (4-6). Here we review the cumulative knowledge of the genetics and clinical presentation of patients with *PROP1* mutations and describe 14 patients who were followed at a single Brazilian center over the past 20 years.

Spontaneous mouse mutants greatly contributed to our initial understanding of CPHD genetics. The Snell and Jackson mice had GH, prolactin and TSH deficiencies. In the early ‘90s, mutations in *Pit1*, which encodes pituitary-specific transcription factor-1, were described in these animals (7,8). This finding quickly led to the detection of mutations in the human homologue gene *POU1F1* (previously known as *PIT1*) with a similar phenotype (9,10). The Ames dwarf mouse, another spontaneous mutant mouse with a similar phenotype, but without mutations in *Pit1,* had the genetic cause established a little while later: mutations in a paired-like homeodomain transcription factor termed ‘Prophet of Pit1’ (*Prop1*) (11). The Ames dwarf phenotype resulted from an apparent failure of initial determination of the *Pit1* lineage, required for the production of GH, prolactin, and TSH, resulting in pituitary dysmorphogenesis and failure to activate *Pit1* gene expression. These results suggested that a cascade of tissue-specific regulators is responsible for the determination and differentiation of specific cell lineages in pituitary organogenesis (11). Finally, in 1998, Wu and cols. reported 4 families in which CPHD was caused by inactivating mutations of the *PROP1* gene in an autosomal recessive pattern (1). These mutations in the human *PROP1* gene resulted in a gene product with impaired DNA binding and transcriptional activation ability. In contrast to individuals with *POU1F1* mutations, patients with *PROP1* mutations also had luteinizing hormone (LH) and follicle-stimulating hormone (FSH) deficiencies (1).

**Figure 1:**
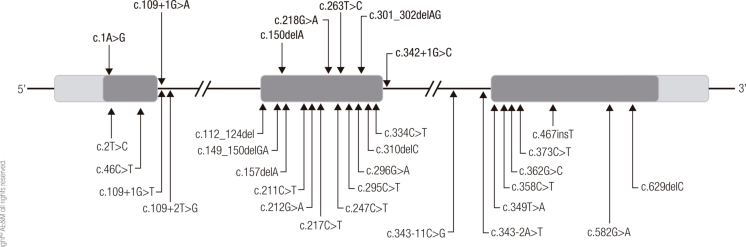
Location of pathogenic variants of the *PROP1* gene. The mutations detected in our cohort are shown in bold type.

*PROP1* (Prophet of Pit1), located in the long arm of chromosome 5 (5q35.3), has 3 exons and encodes a 226-amino-acid-paired–like homeodomain transcription factor (Figure 1) (12). In 1998, Cogan and cols. and Deladoëy and cols. reported a high frequency of the c.301_302delAG (also known as c.296_297delAG) deletion in exon 2 of *PROP1,* which remains the most frequent *PROP1* mutation (13,14).

## CLINICAL CHARACTERIZATION

Since Wu and cols. first described CPHD families with homozygosity or compound heterozygosity for inactivating mutations in the *PROP1* gene, several cases have been reported and more detailed phenotypic characteristics have been described (1).

Patients with *PROP1* mutations do not have an increased prevalence of birth via breech delivery or frequent complications during gestation (15). At birth, in contrast to patients with congenital CPHD caused by other etiologies, neonates with *PROP1* defects lack perinatal signs of hypopituitarism. Mean birth weights and lengths are usually within the normal range, while neonatal hypoglycemia and prolonged neonatal jaundice are rare (15,16).

The phenotype usually includes short stature during childhood and a lack of sexual development at puberty. Distinct phenotypes can be found with hormonal deficiencies progressing with age and asynchronously over time, even among individuals carrying the same genotype (3,17).

### Hormonal deficiencies

The impairment of pituitary production usually follows a pattern. GH and TSH deficiencies are usually present at diagnosis, LH and FSH deficiencies are noted at the onset of puberty, and ACTH deficiency, when it occurs, may develop during follow-up (15).

Short stature is generally the first symptom reported in *PROP1* patients, probably due to combined GH and TSH deﬁciencies. Growth failure usually develops within the ﬁrst year of life (height, -1.5 ± 0.9 SDS at 1.5 years of age) and becomes more prominent later in infancy and early childhood, mainly between the ages of 1.5 and 3 years (-3.6 ± 1.3 SDS at 3 years of age), when parents seek medical assistance (15,16). At diagnosis, the bone age is usually severely delayed (median, 3.3 years) (18).

Although TSH deficiency can present shortly after birth, it usually occurs together or after the onset of GH deficiency and before the age of 20 years (mean age, 6.8 years according to Deladoëy and cols.) (14,17). It is important to note that TSH deficiency can be present in patients with an apparently normal TSH-T4 axis. Bottner and cols. diagnosed most cases early in the clinical course based on insufficient responses to thyrotropin-releasing hormone (TRH) stimulation tests; basal TSH levels were within the normal range and free T4 levels were only slightly decreased (15). Hypothyroidism is usually mild in these cases, in contrast to several patients with *POU1F1* mutations who have severe hypothyroidism and cretinism (19).

Gonadotroph function can also progressively decline and present as a primary or secondary lack of reproductive function. Clinically, it may manifest as a lack of pubertal development, i.e. failure to enter or complete puberty (14,20). There are reports of spontaneous puberty with a posterior decline of gonadotrophic function in Arg120Cys, Phe88Ser, and c.150delA *PROP1* mutations (4,17,21). Impaired gonadotrophic function occurs in the most common *PROP1* mutation (c.301_302delAG).

In 1999, Mendonca and cols. reported on an evolving ACTH deficiency in one patient (3). A corticotrophin deficiency was later confirmed in many but not all patients with *PROP1* deficiency. It appears unlikely that a *PROP1* transcription factor deficiency directly causes ACTH deficiency, but this clinical finding suggests that *PROP1* has some role in corticotroph differentiation or viability. Since adrenal function has been shown to gradually decline over time, even after more than four decades, surveillance is extremely important in all patients with *PROP1* mutations (20,22,23). Furthermore, as GH replacement can increase cortisol metabolism, it is necessary to be aware of the signs of an unveiled adrenal insufficiency (24). The literature review revealed that the mean age at ACTH deficiency diagnosis is 25.3 years (range, 7.4–67 years), confirming previous data demonstrating the emergence of an ACTH deficiency in the third decade of life (1,22).

Among our 14 patients, 3 were born by cesarean section due to obstetrical indications, the median weight was 3435 ± 390 g (SDS + 0.65 ± 0.92); and 2 presented with prolonged neonatal jaundice. Eight of 10 patients for whom term birth weight was available had birthweights according to gestational age above 50^th^ percentile (Fetal Growth Longitudinal Study, World Health Organization) (25). This is similar to another cohort of patients with *PROP1* deficiency (15). This finding contrasts with that of the majority of patients with GH deficiency of the KIGS database (26). At diagnosis (mean age, 16.3 years), the average bone age delay was -6.0 years. Only 1 patient presented with an isolated GH deficiency, while 7 presented with GH and TSH deficiencies. Six patients started follow-up at the post-pubertal period; thus, they were already diagnosed with LH/FSH deficiency. The remaining patients failed to develop spontaneous puberty; in all cases, puberty was induced with sex steroids. Regarding ACTH deficiency, at the first visit, only one patient had a partial ACTH deficiency (35.8 years). Seven patients evolved to ACTH deficiency (mean age at diagnosis, 28 years, range 11.9–52.1 years) (Table 1).

**Table 1:** Clinical features and genetic diagnosis of 14 patients with *PROP1* mutation followed at *Hospital das Clínicas*, University of Sao Paulo Medical School

Patient	Gender	First visit				Birth weight (g)	Final hormone deficiencies	Anterior pituitary lobe	*PROP1* variants

		CA (yrs)	Height		Bone age (yrs)				

			Cm	SD					
1	F	4.5	92.5	-3.1	2.5	3650	GH, TSH, LH/FSH*, ACTH	Hypoplasia	c.263T>C
2	F	6.3	100.0	-2.9	2.0	3420	GH, TSH, ACTH, LH/FSH*	Normal	c.301_302delAG
3	F	6.6	101.1	-3.0	2.5	3780	GH, TSH, LH/FSH*, ACTH	Hyperplasia/hypoplasia	c.301_302delAG
4	F	7.4	103.1	-3.3	3.0	3550	GH, TSH, LH/FSH*	Hypoplasia	c.1A>G/c.263T>C
5	F	12.3	107	-6.1	7.8	2500	GH, TSH, LH/FSH*	Hypoplasia	c.301_302delAG
6	F	12.3	106.4	-6.4	5.5	3700	GH, TSH, LH/FSH*, ACTH	Hypoplasia	c.301_302delAG
7	F	14.9	104.0	-9.4	2.5	NA	GH, TSH, LH/FSH, ACTH	Hypoplasia	c.301_302delAG
8	F	25.8	126.5	-6.0	13.0	3200	GH, TSH, LH/FSH	Hypoplasia	PROP1 complete deletion
9	F	30.3	117.2	-7.5	10.0	NA	GH, TSH, LH/FSH	Hypoplasia	c.301_302delAG
10	F	35.5	119.0	-7.2	13.5	NA	GH, TSH, LH/FSH, pACTH	Hypoplasia	c.301_302delAG
11	F	38.2	136.1	-4.4	-	2780	GH, TSH, LH/FSH*, ACTH	Normal/ microadenoma	c.218G>A/c.342+1G>C
12	M	6.8	106	-2.5	2.7	3450	GH, TSH, LH/FSH*, ACTH	Hypoplasia/ microadenoma	c.1A>G/c.263T>C
13	M	9.5	97.0	-6.0	3.5	3250	GH, TSH, LH/FSH*, ACTH	Hypoplasia	c.301_302delAG
14	M	18.3	123	-7.8	-	NA	GH, TSH, LH/FSH, pACTH	Hypoplasia	c.109+1G>A/c.301_302delAG

CA: chronological age; F: female; M: male; TH: target height; NA: not available. * Patients developed LH/FSH deficiency during follow-up. Patient 6 was born preterm; patients 3 and 5 had neonatal jaundice. pACTH: partial adrenocorticotropin hormone deficiency (basal cortisol level > 5.0 µg/dL and peak cortisol level < 18.1 µg/dL.

The TRH stimulation test performed in 11 patients showed TSH peak levels of 2.9–6.8 mU/L with abnormal increment (<5 mU/L) (3). Prolactin levels were more variable with the peak after TRH stimulation (range, 1.5–30.2 ng/mL). However, in 7 patients, the prolactin peak was below 6.1 ng/mL. Patients with the highest prolactin peak levels also had the highest TSH peak levels. These findings suggest a pituitary rather than hypothalamic defect.

### Neuroimaging

In patients with *PROP1* mutations, neuroimaging of the hypothalamic–pituitary region usually shows a hypoplastic or normal anterior pituitary lobe and a topic posterior pituitary lobe, similar to *POU1F1* patients (27). This contrasts with the majority of patients with CPHD in whom ectopic posterior lobe and other midline defects are commonly detected (21,28).

Changes in pituitary size and morphology can occur over time and were first reported in 1999 (3). An 8.8-year-old girl presented with a diffusely enlarged pituitary gland with a hyperintense signal on T1-weighted magnetic resonance imaging (MRI); the anterior pituitary markedly decreased in size when the patient was 15 years old (height, from 8 mm to 2 mm). An enlarged pituitary gland may be mistaken for a tumor, leading to unnecessary surgery (29). On the other hand, anterior pituitary adenomas have been reported. The surgical removal of the mass revealed an amorphous material but no recognizable cell types on histopathological examination (3,30).

The pituitary can wax and wane in size before undergoing complete involution for reasons yet to be determined (31). Voutetakis and cols. suggested that the mass causing the pituitary enlargement most likely originates from the intermediate lobe (30). This enlargement might result from abnormal development of the anterior lobe and the absence of physiological regression of the intermediate pituitary lobe during organogenesis as was demonstrated by Ward and cols. in Ames dwarf mice (32). According to Ward and cols., the basis of the pathogenesis of pituitary lesions in *PROP1* mutations is the arrest of normal proliferation and migration of the anterior pituitary lobe cells combined with dysmorphic hyperplasia of the dorsal part of the Rathke’s pouch.

Among our cohort of 14 patients, MRI showed a hypoplastic anterior pituitary in 11. The pituitary gland size varied over time from hyperplastic to normal in only 1 patient, while 2 had normal-sized pituitary glands (Table 1). Two patients (patients 11 and 12; Table 1) had pituitary imaging suggestive of microadenomas. Patient 11 presented a small anterior pituitary (height, 3.5 mm) with a hypointense nodular area (3.0 mm, T1-weighted MRI). Patient 12 presented a normal anterior pituitary (height, 5 mm) with a hypointense area (3.2 mm, T1-weighted MRI) suggestive of microadenoma.

## GENETIC DIAGNOSIS

Pathogenic *PROP1* mutations are the most frequent cause of congenital CPHD, with a prevalence of 0.8–64.8% in different countries (2,18). In our center, we screened 29 Brazilian index cases with CPHD and topic posterior pituitary lobe; 52% (15/29) of them carried bi-allelic *PROP1* mutations (5). *PROP1* defects do not lead to midline defects such as ectopic posterior pituitary lobe (EPP) or septic-optic dysplasia. It is noteworthy that the majority of patients with congenital CPHD present with EPP (2).

*PROP1* mutations lead to CPHD in an autosomal recessive inheritance pattern; therefore, the prevalence of these mutations is higher in familial cases (2,5). Among our Brazilian cohort of patients with CPHD and topic posterior pituitary lobe, all 6 familial cases presented with *PROP1* mutations versus only 9 of the 23 sporadic cases (5). Like other recessive genetic disorders, the frequency of *PROP1* mutations is also higher in patients born to consanguineous parents. In our experience, 5 of 7 sporadic cases born to consanguineous parents presented with *PROP1* mutations versus only 4 of 16 sporadic cases born to non-consanguineous parents (5).

Different types of molecular defects reportedly disrupt *PROP1* function, varying from complete gene deletion to frameshift small deletions and insertions and point mutations including missense, nonsense, splicing variants, and mutations affecting the initiation codon (5). Table 2 and Figure 1 summarize all *PROP1* mutations reported to date.

**Table 2:** *PROP1* variants reported to date. Effect on protein and classification according to ACMG guidelines (33). The mutations detected in our cohort are shown in bold type

Mutation	Effect on protein	ACMG classification	Reference
**c.1A>G**	**Initiation codon mutation**	**Pathogenic**	**(5)**
c.2T>C	Initiation codon mutation	Pathogenic	(28)
c.46C>T	p.Arg16Ter	Pathogenic	(40)
**c.109+1G>A**	**Splicing site change**	**Pathogenic**	**(5)**
c.109+1G>T	Splicing site change	Pathogenic	(28)
c.109+2T>G	Splicing site change	Pathogenic	(13)
c.112_124del	p.Ser38Profs*123	Pathogenic	(41)
c.149_150delGA	p.Gly52Aspfs*58	Pathogenic	(42)
**c.150delA**	p.Arg53Aspfs*112	**Pathogenic**	**(36)**
c.157delA	p.Arg53Aspfs*112	Pathogenic	(42)
c.211C>T	p.Arg71Cys	VUS	(43)
c.212G>A	p.Arg71His	VUS	(43)
c.217C>T	p.Arg73Cys	Pathogenic	(12,44)
**c.218G>A**	**p.Arg73His**	**Pathogenic**	**(45)**
c.247C>T	p.Gln83Ter	Pathogenic	(46)
**c.263T>C**	**p.Phe88Ser**	**Pathogenic**	**(4)**
c.295C>T	p.Arg99Ter	Pathogenic	(47)
c.296G>A	p.Arg99Gln	Likely pathogenic	(48)
**c.301_302delAG**	p.Leu102Cysfs*8	**Pathogenic**	**(1)**
c.310delC	p.Arg104Glyfs*61	Pathogenic	(49)
c.334C>T	p.Arg112Ter	Pathogenic	(50)
**c.342+1G>C**	**Splicing site change**	**Pathogenic**	**(5)**
c.343-11C>G	Splicing site change	Likely pathogenic	(49)
c.343-2A>T	Splicing site change	Pathogenic	(12)
c.349T>A	p.Phe117Ile	Pathogenic	(1)
c.358C>T	p.Arg120Cys	Pathogenic	(1)
c.362G>C	p.Arg121Thr	VUS	(51)
c.373C>T	p.Arg125Trp	Pathogenic	(49)
c.467insT	p.Tyr157Leufs*36	Pathogenic	(52,53)
c.582G>A	p.Trp194Ter	Pathogenic	(53,54)
c.629delC	p.Pro210Hisfs*25 (*prolonged protein*)	Pathogenic	(54)

ACMG: American College of Medical Genetics (33); VUS: variant of uncertain significance.

The majority of *PROP1* mutations classified as pathogenic according to the American College of Medical Genetics (33) are located on exon 2 and affect the *PROP1* homeodomain, which is crucial for activating *POU1F1* expression and pituitary organogenesis (34).

The most prevalent *PROP1* mutation is the c.301_302delAG (p.Leu102Cysfs*8), which leads to a premature stop codon at residue 110 (1,13). This frameshift deletion is especially frequent in Eastern Europe, Portuguese, and Latin American patients, probably due to two ancestral founder haplotypes: one that originated from the Baltic Sea area around 2525 years ago and another that originated from the Iberian Peninsula around 583 years ago (35). The c.301_302delAG mutation, the most common genetic diagnosis in Brazilian patients with CPHD and topic posterior pituitary, was present in 9 of 15 index patients with *PROP1* mutations in our group (5).

The second most prevalent mutation is the frameshift 1-bp deletion c.150delA (p.Arg53Aspfs*112) (36), which is probably due to the founder variant that originated from the Belarus region around 1093 years ago (35). Interestingly, we detected this mutation in only one index case in our cohort (5).

Although the known founder effect of the c.301_302delAG mutation in both Portuguese and Spanish populations potentially contributes to a high prevalence of *PROP1* mutations in our cohort, 6 additional *PROP1* mutations were identified in our population. The multi-ethnic origin of the Brazilian population (Amerindian, African, various European, and Asian immigrants) may contribute to these findings (5).

The molecular diagnosis of patients with congenital CPHD impacts the clinical follow-up: patients carrying *PROP1* mutations fail to achieve normal puberty and may develop ACTH deficiency, while those carrying *POU1F1* mutations present with only GH, TSH, and PRL deficiencies. Furthermore, confirming the diagnosis allows genetic counseling for family members and may contribute to the diagnosis of other affected relatives.

Although several algorithms have been designed to guide candidate gene screening by the Sanger method to identify the molecular etiology of congenital CPHD (2,37,38), mutations have been detected using this approach in only a minority of cases (2). In this context, large-scale sequencing (LSS) is a promising approach that sequences large stretches of DNA base pairs, which may include the entire exome (whole-exome sequencing) or multiple candidate genes (in an LSS panel) in a single sequencing run (39). Due to its capacity to simultaneously screen multiple genes, this method may be especially useful when genotype–phenotype correlation is poor or a candidate gene is not suspected (39). Although LSS has great potential for identifying novel genetic causes of rare diseases and expanding the phenotypes associated with known genes, it remains an expensive technique that is not available to all patients. Therefore, in patients with congenital CPHD and topic posterior pituitary who live in areas with a high prevalence of *PROP1* mutations such as Brazilian and some Eastern European cohorts, sequencing *PROP1* using the Sanger method is an adequate first step of a molecular investigation. If no *PROP1* mutations are found, an LSS panel or whole-exome sequencing could be considered.

## CONCLUSIONS

Since its first description 20 years ago, a large expansion of the phenotypes of patients with *PROP1* mutations has occurred. In addition to deficiencies in GH, TSH, PRL, and gonadotropins, some patients develop late ACTH deficiency. Therefore, all patients require permanent surveillance. On MRI of patients with *PROP1* mutations, the pituitary stalk appears intact and the posterior pituitary lobe appears in the normal position, in contrast to the majority of patients with CPHD, who have an interrupted stalk and ectopic posterior pituitary lobe. The anterior lobe in patients with *PROP1* mutations is usually hypoplastic, but it may be normal or even enlarged and can wax and wane in size. Bi-allelic *PROP1* mutations are presently the most frequently recognized genetic cause of CPHD worldwide. Here we classified all reported *PROP1* variants identified to date according to ACMG-AMP guidelines into 29 pathogenic, 2 likely pathogenic, and 2 variants of uncertain significance. The prevalence is higher among offspring of consanguineous parents and familial cases, but sporadic cases can also occur.

Here we summarized the clinical, hormonal, imaging, and genetic characteristics of 14 patients followed at the *Hospital das Clínicas* of University of Sao Paulo Medical School with 7 different *PROP1* mutations, the most diverse in a single unit. *PROP1* mutations are the most frequent cause of autosomal recessive CPHD with topic posterior pituitary lobe.
